# Problematic video game use in adolescents and psychosocial functioning

**DOI:** 10.1192/j.eurpsy.2023.1518

**Published:** 2023-07-19

**Authors:** I. Belabbes, K. Douk, Z. Elmaataoui, H. Kisra

**Affiliations:** 1 Arrazi hospital, Sale; 2 Military hospital mohammed V; 3 Arrazi hospital, Rabat, Morocco

## Abstract

**Introduction:**

Video gaming is an extremely popular leisure activity, with over two billion users worldwide (Newzoo, 2017).

Nevertheless, excessive video game playing exposes to potential dangers. The WHO reminds us that “every gamer should be aware of the time spent on games, especially if their daily activities are affected, as well as any physical or psychological, social and health changes that could be attributed to gaming behaviour.”

**Objectives:**

The aim of our study is to analyse the frequency of video game use, and to determine its relationship with psychological and social functioning and academic performance.

**Methods:**

A cross-sectional study was conducted in the child psychiatry department at Arrazi Hospital in Salé among adolescents aged between 10 and 17 years.

For this, we used:

- A hetero-questionnaire on socio-demographic characteristics, reasons for gambling, type of gambling, satisfaction, self-esteem, ability to make friends and degree of social support, and school results
- The DSM 5 criteria proposed in the appendix for the research
- The K-SADS

**Results:**

Socio-demographic data

- Our study was carried out on a sample of 57 adolescents aged between 10 and 17 years, with an average age of 13.47.
- There was a predominance of males:

Boys: 87.5 , Girls: 17.5

All the adolescents are in school:

Primary 26.3

Middle school 59.6

High school 14 %

Social functioning and academic performance

- 54.4% reported having both real and virtual friends, 29.8% specified that all their friends are virtual, And 15.8% noted that they have no friends
- Academic decline was noted by the parents of 61.1% of adolescents Psychological functioning
- 68.4% reported low self-esteem
- 31.6% reported being dissatisfied with their lives
- 0.5% met the diagnostic criteria for video game addiction

**Conclusions:**

Our study finds that problematic video game use is related to male gender, low academic performance, difficulties with social interactions and also low self-esteem and satisfaction.

Screening for psychiatric co-morbidities and vulnerability factors is essential for the management of this type of pathology.

**Disclosure of Interest:**

None Declared

